# Improving Outcome in Infantile Autism with Folate Receptor Autoimmunity and Nutritional Derangements: A Self-Controlled Trial

**DOI:** 10.1155/2019/7486431

**Published:** 2019-06-18

**Authors:** Vincent Th. Ramaekers, Jeffrey M. Sequeira, Marco DiDuca, Géraldine Vrancken, Aurore Thomas, Céline Philippe, Marie Peters, Annick Jadot, Edward V. Quadros

**Affiliations:** ^1^Center of Autism, University Hospital Liège (CHU), Belgium; ^2^Department of Medicine, SUNY-Downstate Medical Center, Brooklyn, New York, USA

## Abstract

**Background:**

In contrast to multiple rare monogenetic abnormalities, a common biomarker among children with infantile autism and their parents is the discovery of serum autoantibodies directed to the folate receptor alpha (FR*α*) localized at blood-brain and placental barriers, impairing physiologic folate transfer to the brain and fetus. Since outcome after behavioral intervention remains poor, a trial was designed to treat folate receptor alpha (FR*α*) autoimmunity combined with correction of deficient nutrients due to abnormal feeding habits.

**Methods:**

All participants with nonsyndromic infantile autism underwent a routine protocol measuring CBC, iron, vitamins, coenzyme Q10, metals, and trace elements. Serum FR*α* autoantibodies were assessed in patients, their parents, and healthy controls. A self-controlled therapeutic trial treated nutritional derangements with addition of high-dose folinic acid if FR*α* autoantibodies tested positive. The Childhood Autism Rating Scale (CARS) monitored at baseline and following 2 years of treatment was compared to the CARS of untreated autistic children serving as a reference.

**Results:**

In this self-controlled trial (82 children; mean age ± SD: 4.4 ± 2.3 years; male:female ratio: 4.8:1), FR*α* autoantibodies were found in 75.6 % of the children, 34.1 % of mothers, and 29.4 % of fathers versus 3.3 % in healthy controls. Compared to untreated patients with autism (n=84) whose CARS score remained unchanged, a 2-year treatment decreased the initial CARS score from severe (mean ± SD: 41.34 ± 6.47) to moderate or mild autism (mean ± SD: 34.35 ± 6.25; paired t-test p<0.0001), achieving complete recovery in 17/82 children (20.7 %). Prognosis became less favorable with the finding of higher FR*α* autoantibody titers, positive maternal FR*α* autoantibodies, or FR*α* antibodies in both parents.

**Conclusions:**

Correction of nutritional deficiencies combined with high-dose folinic acid improved outcome for autism, although the trend of a poor prognosis due to maternal FR*α* antibodies or FR*α* antibodies in both parents may warrant folinic acid intervention before conception and during pregnancy.

## 1. Introduction

Autism spectrum disorders represent neurodevelopmental disorders characterized by qualitative impairment of communicative and noncommunicative skills, impaired social interaction, and limited interests with stereotypies. Earlier classifications distinguished between infantile autism, Asperger syndrome, childhood disintegrative disorder, PDD-NOS, and Rett syndrome.

So far, no single etiology or common final pathway explaining the features shared by all autism spectrum disorders has been identified, although a number of diverse monogenetic, infectious, toxic or environmental causes have been associated with a minority of cases [1–5].

In the prenatal period, adequate folate delivery to the developing embryo is necessary to prevent the occurrence of neural tube defects (NTD) and possibly other congenital malformations [6]. Maternal folate deficiency increases the risk not only for NTD, but also for autism spectrum disorders (ASD) [7, 8]. Even in the presence of a normal maternal folate status, maternal serum FR*α* autoantibodies directed against the FR*α* localized at the placental barrier were shown to block adequate folate delivery across the placenta, predisposing to intrauterine folate deficiency with consequent congenital malformations or autism spectrum disorders [9–13]. Postnatal development of serum FR*α*-autoantibodies directed against the FR*α* attached to the choroid plexus epithelial cells at the blood-brain barrier causes the so-called infantile-onset cerebral folate deficiency (CFD) syndrome associated with autism in about 1/3 of cases. Subsequent studies also confirmed a high prevalence of serum FR*α* autoantibodies in autism spectrum disorders without neurological deficits, where these FR*α* antibodies were identified in both the child and its parents [9–12, 14–16].

Previous studies on autism suggested increased vulnerability to oxidative stress and decreased methylation capacity as contributory factors [17–20]. However, existing evidence was heterogeneous and inconclusive since many studies were limited by the small size [21]. Further studies postulated mitochondrial dysfunction underlying oxidative stress [14, 15]. Oxidative stress refers to an imbalance between prooxidative factors and antioxidants resulting in abundant production of reactive oxygen species (ROS), being superoxide anions, hydrogen peroxide, and hydroxyl radicals. These ROS possess highly reactive, unpaired electrons capable of initiating a cascade of biochemical reactions with damage to proteins, carbohydrates, fatty acids, lipids, and DNA molecules. Since autistic individuals often manifest feeding difficulties, it is not surprising that excess or multiple deficiencies of vitamins, metals, and trace elements will develop, part of which are essential nutrients and cofactors for intermediary brain metabolism and for antioxidant defences like cofactors of antioxidant enzymes and radical scavengers [21–25]. Thus, in addition to mitochondrial dysfunction, feeding disturbances, and malabsorption in autism may represent alternative mechanisms responsible for oxidative stress due to increased prooxidative factors and/or failing antioxidant defence mechanisms. Another aspect of feeding disturbances deranging nutrient concentrations is the negative impact of these aberrant nutrient concentrations with regard to brain development, nurturing, structure, neurometabolic processes, and regulation of gene expression.

In a previous study we found that the generation of superoxide anions* in vitro* catabolizes 5-methyl-tetrahydro-folate by 75% within one hour, which can be prevented through preincubation with the radical scavenger ascorbic acid [26]. This study also found that KB-cell culture exposure to superoxide anions and hydrogen peroxide reduces cellular folate incorporation mediated by FR*α* or RFC1 transport mechanisms. Thus transmembrane folate passage mediated by these transporters at the placenta and choroid plexus is expected to be impaired in the presence of ROS and predisposes to intrauterine folate deficiency and cerebral folate deficiency.

For these reasons, the first objective of our study design was to identify and correct aberrant nutritional derangements and in particular those markers contributing to oxidative stress mediated by elevated prooxidants and/or deficient antioxidant factors. In addition, we screened serum FR*α* autoantibodies in children with autism and their parents followed by high-dose folinic acid supplements upon finding positive antibody results. We have chosen a self-controlled treatment trial tailored to each individual by the findings of FR*α* antibodies and abnormal nutritional values. In comparison with the CARS evolution of an age- and gender-matched group of untreated patients, we have monitored the CARS at baseline and after two years of treatment where patients served as their own controls.

## 2. Patients and Methods

### 2.1. Participants

Diagnostic investigations included the ADI-R and ADOS tests, Childhood Autism Rating Scale (CARS), developmental and speech assessment, extensive psychological and psychiatric assessment, and observation at school or in the domestic situation. Each patient had a complete history, physical, and neurologic examination and underwent a brain MRI and prolonged EEG registration.

The CARS score is based on the cumulative score obtained on 15 separate items, where a score below 30 indicates absence of sufficient signs and symptoms evocative of autism, a score between 30 and 36 1/2 is compatible with mild to moderately severe autism, and a score from 37 to 60 is compatible with severe autism [27]. Routine investigations included metabolic screening measuring urinary amino acids and organic acids, and creatine and guanidinoacetate excretion. Genetic testing included Angelman syndrome, fragile-X-syndrome, the MECP2 gene defects, chromosome analysis, and array CGH to detect microdeletions or microduplications.

Only patients diagnosed with nonsyndromic infantile autism and without genetic abnormalities were recruited after exclusion of brain abnormalities, intractable epilepsy, and metabolic and recognizable genetic abnormalities or syndromes.

### 2.2. Design of the Self-Controlled Treatment Trial

The self-controlled intervention trial was approved by both the IRB at State University New York and Ethics committee at Liege University Hospital (Protocol: FOL040113). Partial Funding was provided by Autism Speaks (Grant #8202 to EVQ) for measuring folate receptor autoantibodies.

Recruited patients with nonsyndromic infantile autism have been divided into two groups, the untreated patients serving as a reference group (n=84) and the group of treated patients who participated and completed the self-controlled trial (n=82). Both groups have been matched according to age, gender, CARS score, and the FR*α*-antibody profile for child and parents. In the self-controlled treatment study conducted from 2013 to 2018, all autistic patients from both groups underwent a fasting blood drawing for complete blood count (CBC), serum and RBC folate, vitamin B_12_, plasma homocysteine, renal and liver function, thyroid function (TSH, T3, and T4), lactate, CPK, alkaline phosphatase, serum iron, transferrin and ferritin, calcium, magnesium, cholesterol and apolipoprotein B, copper and coeruloplasmin, zinc, manganese, selenium, coenzyme Q10, vitamin E and gamma-tocopherol, vitamin A and beta-carotene, and vitamin D. Serum samples were used to measure antigliadin antibodies and FR*α* autoantibodies of the binding and blocking type in the patients and their parents (see below).

Prior to the study, the age of untreated patients (n=84) varied between 1 and 16.8 years, and their CARS was plotted as a function of age, providing the evolution of CARS with progressing age among untreated patients. Likewise, the CARS of participating patients who completed the self-controlled trial (n=82) was plotted at baseline and after two years of treatment. The age of the latter patients varied between 1 and 15.9 years.

Based upon previous experience from case-control studies where antioxidant deficiencies and serum FR*α* antibodies were found, we outlined an adapted treatment protocol for each individual aimed at correcting nutritional derangements (deficient or excess nutrient) by oral supplements (Table 1). Blood levels were rechecked every 3-4 months during the study period of at least two years and used to adapt supplement administrations accordingly. In addition, oral administration of high-dose folinic acid was started at a dose of 0.5-1 mg/kg/day, if FR*α* autoantibodies came back positive [28]. Folinic acid doses could be increased up to 2 mg/kg/day with a maximum daily dose of 50 mg, if a clinical response did not occur after 6 months [14, 15, 29]. In this self-controlled trial the CARS score was repeated after therapy over two years and compared to the CARS at baseline. We also looked at the changes in CARS scores depending on the initial serum FR*α* autoantibody titers in the child and as a function of the eight different combinations for each family where FR*α* autoantibodies tested negative in the child and both parents, or FR*α* antibodies were only positive in the child, the mother, father, or any combination thereof.

### 2.3. Serum FR*α* Autoantibodies

The assay for both the blocking and binding FR*α* autoantibodies has been described previously. Blocking FR*α* autoantibodies were expressed as pmoles of folic acid blocked from binding to FR*α* per ml of serum and binding FR*α* autoantibodies were expressed as pmoles of IgG antibody per ml of serum [30, 31]. Serum from 30 healthy controls (age range: 1-18 years) and their parents was tested for the presence of FR*α* autoantibodies.

## 3. Results

### 3.1. Untreated Autism Patients

**Figure 1:**
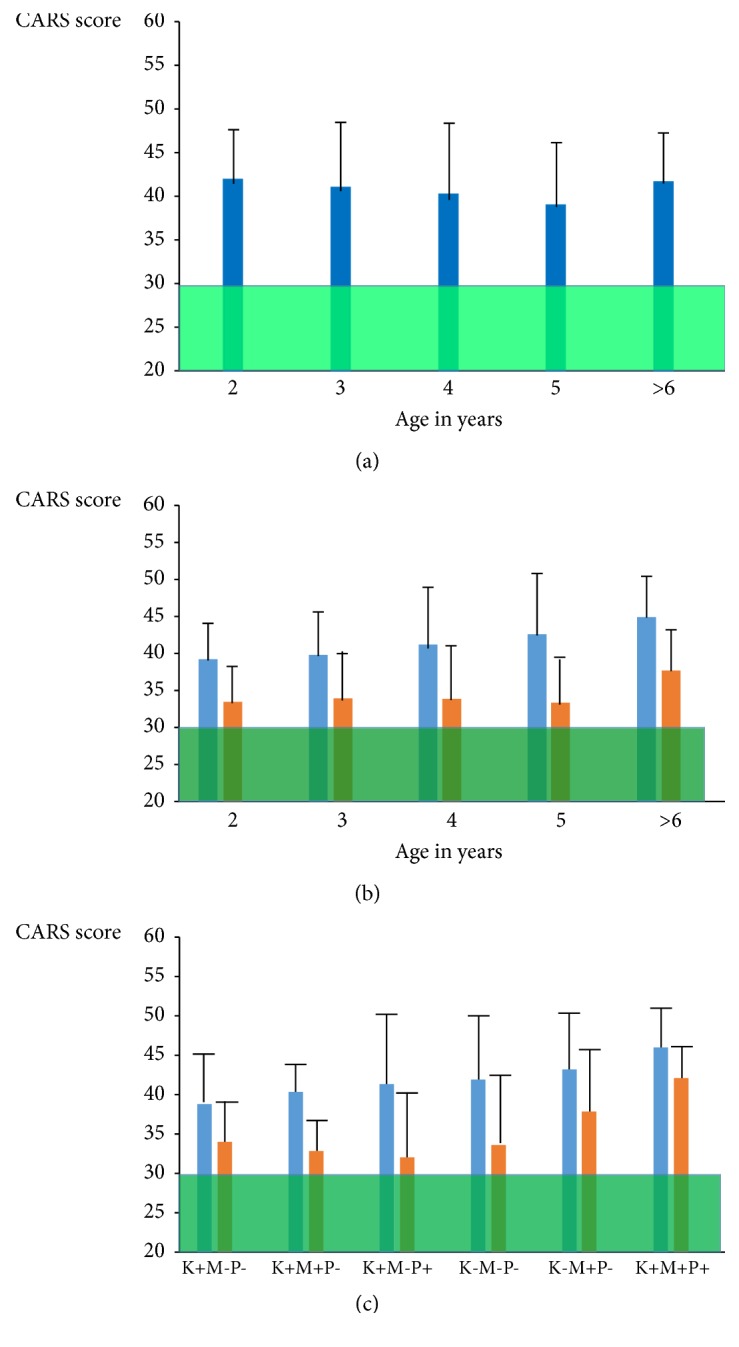
The upper figure (a) shows the plotted CARS with age for 84 untreated patients. The middle figure (b) shows the effect of treatment among 82 treated patients (blue bars represent CARS at baseline and orange bars the CARS after two years treatment). Figure (c) represents the treatment results among different groups with FR autoantibodies in the child (K), mother (M), or father (P).

A group of 84 patients did not give consent to participate in the treatment trial but agreed to have routine laboratory testing as described above. Age (mean ± SD: 4.45 ± 2.62; range: 1-16.8 years), gender (male:female ratio: 5.46:1), and serum FR antibody profiles (71.4 % children testing positive; 30.8 % maternal and 27.4 % paternal serum samples tested positive) matched with the group of 82 who agreed to participate and completed the self-controlled treatment trial. Compared to the baseline CARS scores of the 82 treated patients, the CARS scores for the untreated 84 patients matched and showed no statistical difference in function of each age group ranging from the age group of 2 years up to the age group at or beyond 6 years (Figure 1(a)). The evolution of CARS score for the untreated patient group did not change significantly with advancing age. However, the baseline CARS for the treatment group showed a higher score for the group of children at or beyond 6 years as compared to the baseline CARS assessed for the youngest age groups at 2 and 3 years (see Figure 1(b), Table 2).

### 3.2. Self-Controlled Therapeutic Trial

After informed consent, a total of 115 children diagnosed with severe infantile autism were initially recruited for the self-controlled treatment trial during two years. Ten children were excluded from participation in the study because of underlying syndromes or genetic causes. Parents of one child were first-line cousins; one child suffered from Rubenstein-Taybi syndrome, another child and his mother had the MECP2 duplication, one child had a* de novo* 2p16.3 deletion containing the neurexin I (NRXN I) gene, and one patient was heterozygous for the SLC6A4 p.G56A gene and the SLC29A4 p.D326E gene, encoding the SERT and PMAT proteins, respectively. Copy number variations were found in five other children with a 12p11.21 microdeletion in one child and microduplications in four other children, located at 2q22.2-q23.3, 8p23.3, 16p11.2, and one child with two microduplications at chromosome 15q11.2 and 15q26.3. None of all investigated patients with infantile autism suffered from inborn errors of metabolism, food allergies, or celiac disease.

From 105 children with autism, 82 children (age mean ± SD: 4.4±2.3; range: 1-15.9 years; male/female ratio: 4.8-1) completed the self-controlled trial (Figure 1(b)), whereas 23 children were excluded because of incomplete test results, poor compliance, or failure to attend their follow-up. Serum contained FR*α*-autoantibodies in 75.6 % of all children, while these antibodies were found in 34.1% of the mothers and 29.4% of the fathers. In contrast only 3% healthy controls (age range: 1-18 years) and their parents tested positive for serum FR*α* autoantibodies. Considering the presence or absence of autoantibodies for each trio of child, mother, and father, eight possible combinations of a particular antibody profile existed for each trio. We only found 9/82 families (11%) where FR*α* antibodies tested negative in the child and both parents. In 89% of all families, FR*α*-autoantibodies were present in either the child and/or in one or both parents.

Based on the laboratory results, the treatment protocol for each individual consisted of supplements in combination with high-dose folinic acid (0.5-1 mg/kg/day), if FR*α*-autoantibodies were found. The mean for the CARS of all 82 autistic children at baseline was compatible with severe autism (mean ± SD: 41.34 ± 6.47) and after 2 years of treatment, the CARS declined significantly to a mean value at 34.35, indicating mild to moderate autism (mean ± SD: 34.35 ± 6.25; paired student t-test = 11.72, p<0.0001). In 17 out of 82 children (20.7%), the CARS after treatment dropped below a score of 30, which is consistent with the absence of autistic features. The majority (14/17) of the latter children started treatment before the age of 5 years.

The CARS score lowered significantly following treatment for each age group ranging from 2 years up to 6 years or older. However, as the baseline CARS before treatment increased significantly with advancing age from a mean ± SD of 39.35±5.2 at the age of 2 years towards 45.09±6.46 for children at 6 years or older (unpaired t-test 2.23 and p=0.038), the final CARS outcome remained higher with advancing age despite a similar therapeutic effect on autistic core signs. Therefore, the final outcome became poorer as children grew older and treatment started at a later age beyond 5 years (see Figure 1(b)). However, in the untreated group there was no significant change in the CARS score with advancing age since their mean CARS scores varied between 41.92 at 2 years and 41.75 for the children who were 6 years or older.

We evaluated the effect of treatment for all children, whose parents were negative for FR*α*-antibodies, as a function of FR*α* autoantibody titers. The drop in CARS score (Δ CARS) after treatment was significantly larger for the group of 15 autistic children whose FR*α* autoantibodies of the blocking type were negative or were at a low titer up to 0.44 p mol FR blocked/ml serum (Δ CARS mean ± SD: 7.4±5.22), compared to the 17 children whose FR*α* antibody titers were above 0.44 (Δ CARS mean ± SD: 4.2±3.36; t-test=2.08 and p=0.0455).

Each specific profile among 68 families, where positive or negative FR*α* antibodies were available in the child, mother, or father, was correlated with the CARS before and after treatment with supplements and folinic acid (see Figure 1(c)). Two families are not shown in Figure 1(c) and Table 2 because they represented one single family with only FR*α* autoantibodies in the father and one family where both parents tested positive but the child was negative. In the family where only the father tested positive for FR*α* autoantibodies, the initial CARS of 36 in his daughter dropped to 30 1/2 after correction of her antioxidant and vitamin B_12_ deficiencies. In the family where both parents tested positive, the initial CARS at 42 did not change significantly after 2 years (CARS 41.5) despite treatment.

The results for the other families demonstrated that the presence of positive maternal FR*α* autoantibodies tended to be associated with a higher CARS score at baseline and resulted also in a poorer final outcome after two years treatment. However, comparison of the baseline CARS scores between the group with positive maternal antibodies (N=5) and the group with absent antibodies in the child and parents (N=9) did not reach a statistically significant difference. In particular, when both parents and child tested positive, the high initial CARS score dropped after therapy but this did not represent a highly significant change (p=0.0398). Interestingly, nine families, where the child and both parents had no FR*α* autoantibodies but only had antioxidant or other nutrient deficiencies, had a high mean CARS score at 42.05±6.8, which declined significantly after treatment to a CARS score of 33.88±8.19.

### 3.3. Nutritional Derangements

The results of measured blood parameters among participating children (n=82) in the self-controlled therapeutic trial showed common deficiencies or excess of vitamins, metals, and trace elements well below or above the lower and upper boundaries of reference values established for healthy age-matched controls.

The most frequently encountered deficiencies were vitamin A (65.8%) and vitamin D (62.2%), serum iron (25.6%), ferritin (11%), serum folate (18.3%) and red blood cell folate (18.3%), and moderate apolipoprotein B deficiency (16%). The other deficiencies noted were in decreasing order, gamma-tocopherol (13.4%), manganese (8.5%), selenium (8.5%), coenzyme Q10 (7.3%), beta-carotene (6%), zinc (4.8%), vitamin C (3.6%), and vitamin E (3.6%). Elevated values were found for zinc (11%), selenium (8.5%), manganese (6%), copper (2.4%), and beta-carotene (2.4%), while a disturbed equilibrium between copper and zinc predisposed to an increased copper/zinc ratio, found in 24.3 % of all patients.

Many patients had multiple deficiencies of vitamins, metals, and trace elements simultaneously and received supplements with the advice to adapt their diet with foods rich in deficient factors. Patients with an elevated copper/zinc ratio, elevated metals (zinc, copper, selenium, manganese), or increased beta-carotene, which act as prooxidants, received treatment with high-dose vitamins C and E as radical scavengers within the lipid and aqueous compartments (Table 1).

Blood nutrient levels for elevations or deficiencies were rechecked every 3-4 months, and treatment adapted accordingly. It is interesting to note that the children with autism from 9 families where FR*α* antibodies were absent responded to correction of their deficient nutrient factors and showed a significant decrease of their baseline CARS from a mean ± SD at 42.05 ± 6.8 towards a mean ± SD at 33.88 ± 8.19 after treatment during two years.

## 4. Discussion

Our approach to improve autism outcome following correction of nutrient derangements and FR*α*-autoimmunity has been substantiated by the findings. It also demonstrated that a better outcome after treatment is achieved when FR*α* antibodies are absent or at a low titer (< 0.44 pmol FR*α* blocked/ ml) and if FR*α* autoantibodies are not present in the mother or both parents. Although in the untreated group the baseline CARS remained stable with advancing age, the baseline CARS for the treated group was significantly higher for children at or above 6 years compared to the baseline CARS at the age groups of 2 and 3 years, respectively. This difference for the baseline CARS with advancing age in the two groups is one limitation of the current study and may be attributed to the relative low number of patients in each age group. This difference in baseline CARS was the only parameter that did not match between both groups, whereas age, gender, and FR*α* autoantibody profiles for children and parents matched the untreated with treated groups.

Because the treatment involved correction of both nutrient derangements and FR*α* autoimmunity, the following question remains: which therapeutic strategy has to be indicated as the most effective with respect to clinical improvement? A limited number of autistic children from 9 families where FR*α* autoantibodies were absent in the child and both parents showed a significant improvement in their CARS scores following treatment of identified nutrient derangements. This underscores the important issue to consider not only treatment with high-dose folinic acid supplement for FR*α* autoimmunity, but also to include correction of coexisting nutrient derangements due to feeding problems, which occur frequently in patients with autism spectrum disorders. In this context, it should be stressed that coeliac disease might be responsible for nutrient malabsorption and should be considered among children with autism and specific antibody testing should be included. In our study we identified coeliac disease only in 1 child from the untreated reference group. Feeding problems with consequent nutrient derangements are a common finding in autism and have been attributed to selectivity or aversion to certain foods (particularly fruits and vegetables), combined with gustatory, olfactory, or oral sensory disturbances [21–25].

Several studies suggested that maternal folate deficiency during pregnancy increased not only the risk for neural tube defects but also the risk of autism [7, 8]. A recent study found an association between the use of maternal folic acid supplements during pregnancy and a reduced risk of autism spectrum disorders [32]. Although this and other studies support the association between folate deficiency during pregnancy and an increased risk for autism [7, 8], these studies did not consider the presence of parental FR*α* autoantibodies, identified by us among both mothers and fathers of children with autism [11, 16]. In the event of FR*α* autoantibody detection among mothers, the usually prescribed folic acid dose before and during pregnancy will probably prove largely insufficient [13].

Our self-controlled treatment trial showed that the presence of maternal FR*α* autoantibodies or FR*α* antibodies in both parents tended to be associated with a higher initial baseline CARS score among affected children with autism. Thus, this may explain that the final result and change in CARS score following 2-year treatment was less pronounced as compared to all other groups, although the small number of patients within each group did not allow a profound statistical analysis. These issues will be clarified when more patients will be included into similar treatment trials. Our findings in a minority of 7 out of 68 families (10%) identified no FR*α* autoantibodies in the children whereas FR*α* antibodies could only be detected in the mother (N=5), father (N=1), or both parents (N=1). Although feeding and nutrient problems for each child have to be taken into account, this finding suggests that parental FR*α* antibodies may impair folate transport into oocytes and spermatozoides and also block sufficient folate transport across the placental barrier to the embryo and fetus. Because an adequate folate pool is essential for purine and pyrimidine synthesis, and for mediating epigenetic mechanisms involving DNA methylation and histone modification, the initial embryonic development and subsequent stages of neurodevelopment will rely heavily on availability of adequate folate. Therefore, the risk of autism with its poor prognosis in the offspring associated with parental FR*α* antibodies warrants FR*α* testing among future parents followed by folinic acid intervention before conception and during pregnancy.

The common feeding disturbances associated with autism may provoke oxidative stress due to altered nutritional states where elevated metals (copper, manganese) or beta-carotene act as prooxidants through induction of Fenton chemistry. Nutritional deficiencies of radical scavenging vitamins (vitamins A, C, E, and gamma-tocopherol) as well as metals and trace elements (copper, zinc, manganese, and selenium), being cofactors of antioxidative enzymes, predispose to failing antioxidant defences. Moderate apolipoprotein B deficiency has been encountered in a significant number of autistic subjects and leads to deficient liposoluble vitamins A, D, E, and K. Deficiency of a number of vitamins and coenzyme Q10 necessary for mitochondrial metabolism, will result in mitochondrial dysfunction. Thus, oxidative stress in the brain due to mitochondrial dysfunction, elevated prooxidants, or deficient antioxidants on the one hand and FR*α* autoimmunity on the other hand, represent two independent variables at the basis of autism where correction of each variable showed a clinical response with a decline in the CARS score. Therefore, in addition to treatment for FR*α* autoimmunity [9, 10, 29], specific supplements are required to correct nutritional deficiencies in order to ameliorate intermediary metabolism and to neutralize abundant reactive oxygen species (ROS) deranging brain metabolism and function. As stated above, it appears from our findings in this study that the group of patients, where FR*α* antibodies tested negative in the child and its parents, benefitted only through correction of nutritional derangements as their CARS score dropped significantly.

In our study we also detected deficiencies of serum and red blood cell folate in 18.3 % of all patients.* In vitro* studies have supported the concept of an existing link between oxidative stress and deranged folate homeostasis. In a previous study we found that the generation of superoxide anions* in vitro* catabolizes 5-methyl-tetrahydrofolate by 75% within one hour, which can be prevented through preincubation with the radical scavenger ascorbic acid [26]. This study also found that KB-cells in culture exposed to superoxide anions and hydrogen peroxide reduces cellular folate incorporation mediated by FR*α* or RFC1 transport mechanisms. Thus transmembrane folate passage mediated by these transporters at the placenta and choroid plexus is expected to be impaired in the presence of ROS and predisposes to intrauterine folate deficiency and cerebral folate deficiency.

ROS predispose to nitrosative stress with peroxynitrite formation within neurons expressing neuronal NO-synthase leading to neuronal dysfunction and apoptosis [33–37].

**Figure 2:**
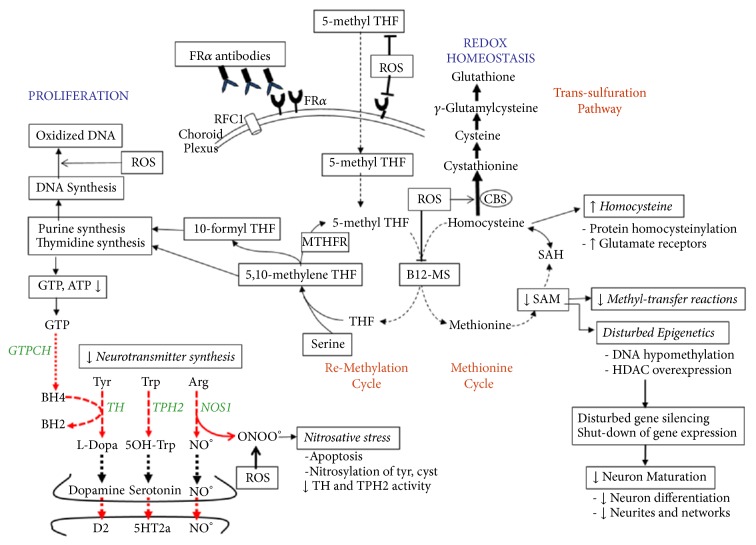
Pathophysiology of autism based on our findings showing the impact of reactive oxygen species (ROS) at different levels of intermediary metabolism and the consequences of brain 5-methyl-tetrahydrofolate (5-methyl THF) deficiency due to FR*α* autoimmunity. ROS inhibits B_12_-methionine synthase (B_12_-MS) activity and stimulates cystathionine-beta-synthase (CBS) activity, shifting the homocysteine accumulation from the methionine cycle into the transsulfuration pathway with increased production of the natural antioxidant glutathione. Superoxide anions also react with NO at the level of NO-synthase (NOS1) to form peroxynitrite instead of NO, which predisposes to apoptosis and nitrosylation of tyrosine and cysteine. Nitrosative stress affects activity of tryptophan- (TPH2) and tyrosine hydroxylases (TH), the rate-limiting enzymes for serotonin and dopamine synthesis. In addition ROS catabolize 5-methyl-THF and impair folate uptake and transcellular transport across the choroid plexus and placental barriers due to interaction with FR*α* and RFC1 folate transporters. FR*α* autoantibodies also impair folate transport to the fetus and brain and predispose to brain folate deficiency with reduction of SAM production and SAM-dependent methyl-transfer reactions, reduced purine and thymidine synthesis with diminished GTP and BH4 production, which is the common cofactor of the enzymes TH, TPH2 and NOS1. Reduction of the activated methyl-group donor SAM down regulates DNA methylation and affects posttranslational modifications of histones (methylation and trimethylation of histones), thereby impeding the homeostatic balance between gene transcription and silencing. In addition folate deficiency is accompanied by overexpression of histone deacetylases, which further leads to abnormal gene silencing. The shutdown in expression of specific sets of genes will affect neuronal growth, pruning and differentiation. Abbreviations: GTPCH: GTP-cyclohydrolase I; cyst: cysteine; tyr: tyrosine; MTHFR: methylene-tetrahydrofolate reductase; RFC1: reduced folate carrier-1.

Thus,* in vivo* superoxide anion generation promotes formation of peroxynitrite in NO-synthase positive neuronal networks (Figure 2). Neuronal folate depletion will further compromise folate-dependent* de novo* purine synthesis with adenosine and guanosine production. The consequent low guanosine triphosphate (GTP) synthesis, which represents the substrate for the rate-limiting enzyme GTP-cyclohydrolase I, will reduce tetrahydrobiopterin (BH4) production [38]. The low BH4 acting as cofactor for tryptophan hydroxylase, tyrosine hydroxylase, and NO-synthase will reduce these enzyme activities and diminish the production of serotonin, dopamine and NO. Moreover, in the presence of low BH4, NO-synthase will shift its enzymatic activity and start to produce the nitrosyl radical peroxynitrite instead of NO [35, 39–41]. Postmortem brain tissue studies have confirmed the presence of biomarkers for oxidative and nitrosative stress in autistic brains, because in cortical brain areas and cerebellum high concentrations of 8-hydroxy-deoxy-guanosine and 3-nitrotyrosine were found [42, 43]. Although the present study did not document disturbed metabolism of pterins, neurotransmitters, and NO, the well-known comorbidities associated with autism like ADHD, anxiety, obsessive-compulsive disorder, and sleeping problems support this hypothesis.

The other consequence resulting from diminished purine synthesis associated with brain folate depletion will be the low adenosine production as substrate for ATP, which can be expected to lead to mitochondrial dysfunction and also to inadequate maintenance of the mitochondrial DNA pool.

Further consequences of specific brain nutrient deficiencies predispose to oxidative stress and can affect the function of key enzymes and intermediary metabolism and predispose to alterations of DNA structure and function. Peroxynitrite inactivates the rate-limiting enzymes for serotonin and dopamine synthesis being tryptophan- and tyrosine hydroxylases via sulfhydryl oxidation at their enzyme substrate binding sites, where the protein is rich in cysteine residues [44–46]. Peroxynitrite will also nitrosylate protein tyrosine residues, but this exerts a minimal effect on enzyme activity [44]. In addition, oxidation induces neuronal tryptophan hydroxylase 2 aggregates through disulfide cross-linking [46]. Thus, generation of ROS and peroxynitrite, enhanced by folate depletion, reduces production of dopamine, NO, and in particular serotonin, which is supported by previous findings of low serotonin production known to affect about 1/3 of autistic patients [38, 47].

Exposure to oxidative stress leads to multiple adaptive mechanisms with inhibition of B_12_-dependent methionine synthase activity, which has a negative impact on the methionine cycle with reduced SAM production, resulting in failure of >100 methyl-transfer reactions [48, 49]. Simultaneously, oxidative stress increases the activity of cystathionine-beta-synthase which shifts homocysteine away from the methionine cycle towards the transsulfuration pathway to increase cysteine and glutathione synthesis as adaptive mechanisms to elevated oxidative stress [50]. In this context, low brain methyl-tetrahydrofolate availability due to FR*α* autoimmunity further inhibits methionine synthase activity and will aggravate the shift from the methionine cycle towards the transsulfuration cycle. Thus, oxidative stress will cause compensatory increases of the antioxidant glutathione and will downregulate the cellular methylation capacity.

ROS also reacts directly with DNA purine and pyrimidine components and causes DNA strand breaks, as reflected by increased oxidized DNA damage in lymphocytes. Moreover, ROS can induce oxidative DNA damage at the level of methylated CpG islands near gene promoter sites resulting in various mutations including C→T transitions, G→T transversions and CG→TT tandem mutations. In addition, ROS can convert guanosine to 8-oxo-guanosine and 5-methyl-cytosine towards 5-hydroxymethyl-cytosine [51–54]. These modified methyl-CpG sequences due to oxidative stress lead to functional loss of methylated CpG islands acting as recognition sites for methyl-CpG binding proteins (MBP), normally required to recruit histone deacetylases for chromatin condensation and gene silencing [54]. Thus, oxidative stress not only causes gene sequence alterations but also results in epigenetic changes with failure of methylated gene inactivation.

Because FR*α* autoantibodies block transport of methyl-tetrahydrofolate across the placenta to the fetus and the choroid plexus barrier to the brain, the consequent brain methyl-tetrahydrofolate depletion diminishes production of sufficient SAM as the activated methyl donor in over 100 methylation reactions in the brain, including the DNA-methyl-transferases which transfer methyl groups to gene CpG promoter sites which serve as recognition sites for methyl-CpG binding domain proteins (MBD) and histone deacetylases required for gene silencing. In this context, the combination of FR*α* autoimmunity and oxidative stress act independently or cooperate to derange epigenetic mechanisms controlling the orchestration of activation and inactivation of specific genes during neuronal development and differentiation processes.

Experiments using rat H19-7 hippocampal cell lines have shown that folate-deficient neurons have decreased proliferation rates, abnormal cell polarity, and migration, as well as failure of differentiation processes such as neurite outgrowth, and expression of glutamate receptors on dendrites, leading to abnormal synaptic function and plasticity [55]. Folate deficiency in hippocampal neuron cultures leads to decreased SAM concentrations and overexpression of histone deacetylases (HDAC 4,6,7). Histone deacetylase overexpression will consequently lead to repression of multiple developmental genes that are normally activated during specific developmental phases [56, 57]. Another consequence of folate deficiency in these hippocampal neuron cell lines is homocysteine accumulation with formation of homocysteinylated proteins (the motor proteins dynein and kinesin, among others), leading to aggregation and dysfunction of key neuronal proteins affecting normal development with possible long-lasting consequences [55, 58–61].

The consequences of folate deficiency affecting brain development may be more prominent in autistic children from mothers with folate deficiency or the presence of maternal FR*α* autoantibodies during pregnancy. Our finding of a higher initial baseline CARS score and less favorable outcome in these children confirms this hypothesis. In summary, the treatment response will be influenced in a negative fashion by the presence of maternal FR*α* autoantibodies, by late-onset treatment associated with a higher initial CARS score and in the event of elevated antibody titers. Paternal FR*α* antibodies may also influence the outcome and need to be further investigated, because we only identified one family.

## 5. Conclusion

In the pathogenesis of low-functioning autism, feeding disturbances predisposing to oxidative stress and acquisition of folate receptor autoantibodies during the pre- or postnatal period appear to play an important role by affecting intermediary metabolism and potentially deranging epigenetic control mechanisms. Early detection and appropriate therapeutic intervention is postulated to reverse core features and improve outcome.

## Figures and Tables

**Table 1:** The treatment protocol for the self-controlled treatment trial based upon abnormal biochemical findings and FR*α* autoantibodies.

*Abnormal biomarker*	*Daily oral supplement dosage*
Zinc deficiency	0.15-0.25 mg/kg zinc-sulfate
Selenium deficiency	3-5 *µ*g/kg sodium-selenite
Manganese deficiency	5-10 mg/kg Vitamin C, 20 IU/kg Vitamine E, with 1 coffespoon Soya oil at night.
Manganese excess	idem
Heavy metal excess (Cu, Al, Hg, Pb)	idem
Raised copper/zinc ratio	idem
Bèta-carotene excess	idem; limit foods rich in bèta-carotene
Vitamin A deficiency	600-1500 *µ*g
Vitamin D (25-hydroxy-D)	10 *µ*g or 400 IU
Vitamin C deficiency	5-10 mg/kg Vitamine C (maximal 500mg)
Ubiquinon-10 deficiency	2 mg/kg co-enzyme Q10
Vitamin E deficiency	20 IU/kg
Gamma-Tocopherole deficiency	1 coffeespoon soya, corn or sesame oil
Bèta-carotene deficiency	Consume tomato or carot juices
Serum folate deficiency	0.5 mg/kg folinic acid
RBC folate deficiency	0.5 mg/kg folinic acid
Apolipoproteine B deficiency	Supplement vitamins A D E, and vitamine K in case of secondary coagulation disorder
FR-alpha antibodies	Start with 0.5-1 mg/kg folinic acid daily;
	Increase to 2 mg/kg daily without a clinical response after six months. Maximum daily dose 50 mg.

**(a):** 

Age	N	CARS score
1	1	36.5
2	20	41.92 ± 5.4
3	21	41.12 ± 6.7
4	21	40.33 ± 7.56
5	9	39.05 ± 4.07
≥ 6	12	41.75 ± 4.25

**(b):** 

Age	N	CARS at baseline	CARS after treatment	paired t-test	p value
		( Mean ± SD)	( Mean ± SD)		
1	1	38	38		
2	10	39.35 ± 5.2	33.6 ± 4.9	3.31	0.009
3	31	40.01 ± 5.8	33.96 ± 5.9	7.79	<0.0001
4	19	41.37 ± 6.7	34 ± 7.9	4.66	0.0002
5	10	42.8 ± 8.2	33.55 ± 5.5	4.99	0.0007
≥ 6	11	45.09 ± 6.46	37.7 ± 5.15	6.02	0.0001

**(c):** 

FR*α* antibody profile			N	CARS at baseline	CARS after treatment	paired t-test	p value
Child	Mother	Father		( Mean ± SD)	(Mean ± SD)		
+	-	-	23	39,06 ± 6,17	34,28 ± 4,21	4.86	<0.0001
+	+	-	11	40,54 ± 3,71	33,14 ± 4,42	5.64	0.0002
+	-	+	11	41.5 ± 9.08	32.32 ± 7.6	4.49	0.0012
-	-	-	9	42.05 ± 6.8	33.88 ± 8.19	7.6	<0.0001
-	+	-	5	43.5 ± 6.5	38 ± 7.06	3.41	0.027
+	+	+	7	46.14 ± 4.8	42.21 ± 3.8	2.61	0.0398

## Data Availability

The data used to support the findings of this study are available from the corresponding author upon request.

## Abbreviations

ADI-R: Autism Diagnostic Interview-Revised
ADOS: Autism Diagnostic Observation Scale
B_12_-MS: B_12_-methionine synthase
BH4: Tetrahydrobiopterin
CARS: Childhood Autism Rating Scale
CBS: Cystathionine-beta-synthase
CFD: Cerebral folate deficiency
cyst: Cysteine
FOLR1: Folate receptor alpha gene
FR*α*: Folate receptor alpha
GTPCH: GTP-cyclohydrolase I
LDL: Low-density lipoprotein
5-Methyl THF: 5-Methyl-tetrahydrofolate
MTHFR: Methylene-tetrahydrofolate reductase
NO: Nitric oxide
NOS1: NO-synthase
NTD: Neural Tube Defect
PDD-NOS: Pervasive developmental disorder, not otherwise specified
RFC1: Reduces folate carrier-1
ROS: Reactive oxygen species
SAM: S-adenosyl-methionine
TH: Tyrosine hydroxylase
TPH2: Neuronal tryptophan hydroxylase
tyr: Tyrosine
MTHFR: Methylene-tetrahydrofolate reductase.
